# The Conundrum of Antidepressant-Induced Anhedonia: A Blended Patient–Psychologist Perspective

**DOI:** 10.1177/23743735251346666

**Published:** 2025-05-27

**Authors:** Nicholas Norman Adams

**Affiliations:** 1 School of Health, 1018 Robert Gordon University, Ishbel Gordon Building, 1018 Robert Gordon University, Aberdeen, UK

**Keywords:** anhedonia, patient author, treatment-resistant depression, MDD, MAOI

## Abstract

This article constitutes a Patient Perspective, grounded in lived experience, its primary aim is to enhance awareness of antidepressant-induced anhedonia by providing experience-based insights, relevant to clinicians, researchers, and caregivers. My own experiences with treatment-resistant depression-anxiety have been significant and long-lasting. In my 22-year-plus journey of illness experience—and having taken over 23 antidepressant medications—emotional-blunting, anhedonia, and mania have all, at times, been side-effect-related factors. This work explores the conundrum of antidepressant-induced anhedonia, developing an in-depth patient perspective useful for mental health practitioners, psychiatrists, psychologists, and for wider formal professional and informal nonprofessional caring actors. I write this via a reflexive lens as a long-term mental health patient, while also recognizing my dual-positionality as a Chartered Psychologist and an academic with a PhD working in the field of mental health. Thus, my dual-perspective provides a unique lens useful for translating the patient experience to a wider caregiving audience: fostering understanding and deepened awareness of the anhedonia experience. Implications for patient care are discussed.

## Introduction

Anhedonia refers to a diminished capacity to derive pleasure. It is a key characteristic of depression and other psychiatric “disorders”^1,2^). Current thinking links anhedonia to dysregulations in dopamine transmission.^3^ While numerous investigations have focused on the ability of antidepressants to counteract anhedonia, little research explores their potential to trigger anhedonia over long-term use. This may be due to underreporting of anhedonia, or limited recognition of this possibility within clinical settings.

Speaking to the limited existing research, Read and Williams^4^ conducted a large-scale international survey exploring adverse antidepressant effects—identifying emotional-blunting as a frequently reported side-effect. Similarly, Goodwin et al^5^ surveyed patients undergoing antidepressant treatment and found that emotional-blunting was commonly reported, negatively affecting emotional engagements and subjective well-being. Jawad et al^6^ conducted a scoping review investigating which antidepressants may contribute to emotional-blunting. While not universally experienced, emotional-blunting was observed in a subset of patients; the work spotlighting urgent requirements for further research. Akin to others, Marazziti et al^7^ also interrogated emotional-blunting as a potential consequence of long-term antidepressant use, centering concerns regarding the impacts of subjective anhedonia experiences upon patients’ emotional well-being. Finally, Peters et al^8^ analyzed data from 3 randomized controlled trials, uncovering emotional-blunting as occurring among patients treated with Bupropion and selective serotonin reuptake inhibitors (SSRIs). Their study suggests that while emotional-blunting is a recognized side-effect of multiple antidepressants, certain drug classes may be more likely to cause anhedonia versus others.

## Perspectives Practical: The Conundrum of Antidepressant-Induced Anhedonia

In my own experience, anhedonia is both a symptom of major depressive disorder (MDD) and a side-effect of antidepressant medications. I encountered anhedonia initially during my first spell of MDD which occurred in my late teens. I discuss this in detail elsewhere.^9^ Then, this anhedonic “emotional-deadness” was a symptom of MDD. However, over time, and after being treated for MDD with a variety of different medications from different medication classes throughout my life^a^, I have come to recognize that anhedonia can readily present as a side-effect of medications as well as a symptom of depressive illness itself. This is not the platform to debate the neurobiological mechanisms by which antidepressants may cause anhedonia—indeed, I write this article from a patient perspective and I am not qualified to delineate a comprehensive argument for this. But, theoretically, I understand the SSRI-mediated mechanism to be linked to medication-mediated reductions in dopamine, and—in my present situation—in the case of monoamine oxidase inhibitor medications—the anhedonia-promoting mechanism to be linked to a downregulation of dopamine receptors over time.

The most immediate challenge when one who is taking antidepressant medication long-term experiences anhedonia is the conundrum of identifying whether the presence of anhedonia signals a return of depressive state: that is, the antidepressant medication(s) is ceasing to work and/or depression is increasing to eclipse the therapeutic ability of medication, or conversely, whether anhedonia is itself a side-effect arising from long-term high-dose antidepressant therapy. This ambiguity compounds the complexities of depressive illness, adding an additional layer of frustration to already negative feelings, and blurring the patient's ability to develop a concrete sense-making of their illness and emotional identity.

As I write this I am experiencing anhedonia, and have been for several months. This is getting worse. The present issue I face is that the 600 mg of Moclobemide I take daily is likely causing this. But—I recognize that when I began Moclobemide over 2 years ago, I was in such a depressive state that I was almost at my end-point. As such, Moclobemide is likely lifting MDD by blunting depressive feelings but is equally flattening emotional response so as to also blunt any positive feelings. Understanding this catch-22 is critical for mental health practitioners when considering issues of psychiatric medication compliance, so—I will explain this here: my options are to discontinue Moclobemide, knowing that this may result in a lessening (or indeed removal) of anhedonia. Yet, such discontinuation is also—quite frankly—a terrifying potentiality, given that this may also result in a return of depressive mood, which is worse than anhedonia.

To summarize the unique contribution of me sharing this highly personal perspective: most existing literatures treat depression and anhedonia as comparable constructs of emotion, suggesting that if one is experiencing an absence of pleasure, they may also be depressed. Indeed, such thinking is validated in diagnostic trends for MDD.^10^ However, I argue the opposite, that such states are distinct. In my own present case, experiencing anhedonia—while distressing—is preferable to experiencing a return of MDD. Thus, experiencing emotional-blunting, low-affect, and detachment, is paradoxical—existing as both a depression-sparing state in one sense, while also being inherently a depressing experience, this itself characterized by an inability to feel pleasure. Thus, when considering my own sense-making, anhedonia is distinct from MDD, rather than being a similar or close-associated construct of being. To draw comparisons, anhedonia is less painful, less immediate, less constraining, and debilitating, but marked with a unique sense of “missing out,” “being numb,” and existing in a state of derealization/depersonalization, while MDD exists as a different experience: a blanket of hopeless crushing numbness, squeezing the life from you, and forcing you to succumb to a state of withdrawal, recession, and absence of the characteristics typical of life.

## Recommendations

I posit that this perspective is useful; providing valuable insight for mental health practitioners supporting individuals experiencing anhedonia, particularly in the context of long-term antidepressant use. Traditionally, anhedonia has been regarded primarily as a symptom of MDD—underpinning assumptions that presence signals a recurrence of depression. However, as I illustrate using my own experiences—anhedonia may also exist as a side-effect of medication, rather than as an indication of worsening depressive mood. This distinction is of critical importance in clinical practice, as it challenges the conventional belief that the inability to experience pleasure is inherently tied to depression. For practitioners, my personal perspective spotlights a requirement for more complex approach to care. It is important to carefully assess whether anhedonia is indicative of ineffective/waning antidepressant effects or whether anhedonia has emerged—gradually—as a side-effect of medication itself. This differentiation can be complex, as emotional-blunting caused by medication may, paradoxically, be more tolerable for some patients than the risk of a severe depressive relapse, and thus, patients may be more reluctant to discuss anhedonia experiences despite their distressing nature for fear of clinicians instigating medication-switching, or suggesting withdrawal of otherwise beneficial antidepressant therapy. Recognizing anhedonia as a distinct phenomenon is therefore crucial in ensuring a thoughtful and patient-experience-informed approach to treatment-planning that explores how emotional-blunting is perceived and made sense of by individuals.

However, it must also be noted that a key challenge in interpreting the experience of antidepressant-induced anhedonia is the issue of reverse causality—namely, clinicians identifying whether anhedonia arises as a symptom of (returning) depression itself or as a side-effect of pharmacological treatment. This diagnostic ambiguity complicates both clinical assessment and therapeutic decision-making. While this account is grounded in long-term lived experience, it is salient to recognize that distinguishing between the effects of medication and the natural course of illness remains a complex clinical task. However, emerging evidence indicates that anhedonia may exhibit unique neurobiological and experiential features, which differentiate it from the core symptoms of depression.^2,11,12^

Argyropoulos and Nutt^11^ contend that anhedonia—especially when accompanied by psychomotor retardation—represents a distinct facet of depression, distinct from mood and somatic symptoms. They highlight neurochemical evidence linking anhedonia to dopamine system dysfunction, in contrast to the serotonin-related basis of mood symptoms. The authors advocate for the use of dopamine-targeting treatments to address anhedonic symptoms more effectively. Similarly, Wu et al^12^ examine anhedonia as a core but clinically and biologically distinct component of MDD. They posit that individuals with marked anhedonia often face more severe episodes and greater risk of suicidality, suggesting a poorer prognosis. Biologically, they posit that such patients may show raised inflammatory markers, metabolic irregularities, and brain-derived neurotropic factor (BDNF) hypermetabolism, alongside neuroimaging evidence of changes in subcortical, cortical, and limbic brain regions. The review supports the perspective that anhedonia differs meaningfully from other depressive symptoms, underscoring the importance of tailored treatment approaches.

Speaking to the discontinuation of established antidepressant medications, it is also important to acknowledge *rebound phenomena*. This can occur when the symptoms being treated by antidepressants worsen or return after discontinuation or prolonged use. This paradoxical effect can lead to increased emotional dysregulation or heightened depressive symptoms, making it difficult to distinguish between medication side-effects and the natural progression of the illness, particularly when attempting to differentiate MDD symptoms and anhedonia.^13,14^

Further, it is also possible that antidepressants modify the brain's pleasure threshold over time, contributing to diminished emotional responses.^15^ Investigating this through controlled observational studies comparing the short-term and long-term effects of antidepressants could offer valuable insights into whether the long-term use of medication exacerbates or alleviates emotional dysregulation and would be a valuable future study regarding linking anhedonia to long-term (and high-dose) antidepressant treatment.

Concurrent to the above points, the personal perspective I share also reveals the significant emotional and psychological toll of prolonged anhedonia. Unlike MDD, which may present as an all-consuming despair, anhedonia manifests as a gradually building but equally distressing sense of detachment, depersonalization, and an overarching feeling of “missing out” on life. For clinicians, it is important to acknowledge and encourage patients to articulate and discuss the distress that anhedonia may be causing, even in the absence of a conventionally defined depressive episode. Effective support should include validating the patient's experiences, exploring potential (coproduced with patients) adjustments to treatments, and working collaboratively to strike a balance between symptom management and emotional engagement. Furthermore, this account underscores the importance of shared decision-making in psychiatric care. The fear of discontinuing medication, coupled with the uncertainty of whether anhedonia or depression poses a greater risk, presents an anxious dilemma for many patients. Practitioners can play a key role in facilitating open discussions around these concerns, carefully delineating the potential benefits and drawbacks of altering medication, openly answering questions, and considering therapeutic strategies that might help alleviate anhedonia without compromising overall mental stability or inviting a recurrence of MDD.

## Conclusion

Understanding anhedonia as a complex and multifaceted experience—rather than simply a marker of depressive relapse—allows for a more patient-centered approach to care. Recognizing the patient-paradox of anhedonia experiences enables clinicians to better support individuals in balancing stability with the need for experiencing emotional richness. My perspective, shaped by both lived experience and my knowledge as a psychologist, highlights the challenges of distinguishing between anhedonia as a symptom of depression and as a side-effect of medication. Having navigated treatment-resistant depression-anxiety for over 22 years and trialed more than 23 antidepressants, I have witnessed firsthand how medication can alleviate depressive symptoms while simultaneously dulling emotional responses. This dilemma forces patients to weigh the benefits of stability against loss of emotional depth, rendering treatment decisions deeply complex. Through acknowledging these challenges, mental health practitioners may adopt more multifaceted approaches—validating the distress of anhedonia while promoting shared decision-making. Greater awareness of anhedonia experiences bridges the gap between clinical understanding and patient reality, ultimately fostering more compassionate and effective care.

## Notes

a. Treatment has included SSRIs, SNRIs, TCAs, atypical medications, adjunctive medication therapy, combined antidepressants, Reboxetine, Agomelatine, Ropinirole, and eventually, MAOIs. My current medication is maximum-dose Moclobemide (a R-MAOI: Reversible Inhibitor of Monoamine Oxidase).
